# Visual-Acoustic Sensor-Aided Sorting Efficiency Optimization of Automotive Shredder Polymer Residues Using Circularity Determination †

**DOI:** 10.3390/s19020284

**Published:** 2019-01-12

**Authors:** Jiu Huang, Chaorong Xu, Zhuangzhuang Zhu, Longfei Xing

**Affiliations:** School of Environment and Spatial Informatics, China University of Mining & Technology, Xuzhou 221116, China; crxu@cumt.edu.cn (C.X.); zzzhu@cumt.edu.cn (Z.Z.)

**Keywords:** sensor-aided sorting, automotive shredder residues, circularity determination, particle size distribution

## Abstract

To reduce the emissions and weight of vehicles, manufacturers are incorporating polymer materials into vehicles, and this has increased the difficulty in recycling End-of-Life vehicles (ELVs). About 25–30% (mass) of an ELV crushed mixture is the unrecyclable material known as automotive shredder residues (ASRs), and most of the vehicle polymers are concentrated in this fraction. Thus, these vehicle polymers are conventionally disposed of in landfills at a high risk to the environment. The only way to solve this problem is through the development of a novel separation and recycling mechanism for ASRs. Our previous research reported a novel sensor-aided single-scrap-oriented sorting method that uses laser-triangulation imaging combined with impact acoustic frequency recognition for sorting crushed ASR plastics, and we proved its feasibility. However, the sorting efficiencies were still limited, since, in previous studies, the method used for scrap size determination was mechanical sieving, resulting in many deviations. In this paper, a new method based on three-dimensional (3D) imaging and circularity analysis is proposed to determine the equivalent particle size with much greater accuracy by avoiding the issues that are presented by the irregularity of crushed scraps. In this research, two kinds of commonly used vehicle plastics, acrylonitrile-butadiene-styrene (ABS) and polypropylene (PP), and their corresponding composite materials, acrylonitrile-butadiene-styrene/polycarbonate (ABS/PC) and polypropylene/ethylene-propylene-diene-monomer (PP/EPDM), were studied. When compared with our previous study, with this new method, the sorting efficiency increased, with PP and PP/EPDM and ABS and ABS/PC achieving about 15% and 20% and 70% and 90%, respectively. The sorting efficiency of ASR polymer scraps can be optimized significantly by using sensor-aided 3D image measurement and circularity analysis.

## 1. Introduction

With the rapid development of the global vehicle industry, both the worldwide production and ownership of automobiles have increased steadily over the last 20 years, making the automotive industry one of the biggest in the world [1,2]. In China, for example, vehicle production and sales both reached 28 million in 2016 [3] and car ownership is expected to increase to 280 million by 2020 [4]. However, at the same time, the automobile industry is facing numerous challenges, such as energy consumption, greenhouse gas emission, and, finally, waste disposal of End-of-Life vehicles (ELVs) [5]. To lessen the consumption of petrol and its associated emissions, lighter vehicles are now manufactured and driven worldwide. Vehicle weight reduction relies on the replacement of some of the metal components with polymer materials [6,7]. However, as opposed to polymers, metals can be recycled conveniently. Thus, the use of polymers in automobile manufacturing has created many difficulties for ELV recycling.

In ELVs, the metal fraction represents, on average, about 70–75% (by mass) of the total weight, which can be recycled effectively using conventional methods. The remaining 25–30% is called automotive shredder residues (ASRs), which are generated during the ELV shredding process. ASRs are extremely heterogeneous, as they are a mixture of ferrous and nonferrous metals, plastics, rubbers, fibers, woody materials, etc. [8,9]. Thus, ASRs are often disposed of in landfills or are used for energy recovery, which involves a high risk of contamination. Hence, ASRs are classified as hazardous materials and they can cause serious environmental pollution [7,9,10]. ASRs contain approximately 40% of the polymers included in vehicles [11,12].

The leading producers of vehicles are in Europe, North America, and Japan. There has been legislation introduced to address the problem of ELV recycling. For example, Europe established the ELV Directive 2000/53/EC, which set a target of 95% (by mass) for the reuse or recovery rate of materials in ELVs, including a deadline of 1 January 2015, by which time the material recycling rate should not be lower than 85% (by mass) [5,7,10,11,12]. The most direct and efficient approach to achieving these targets and fulfilling the directive’s requirements is to increase the recycling rate of polymers, especially the plastics in ASRs. However, conventional technologies cannot support this work. Unlike metals, most of the polymers that are used in vehicles are insensitive to force fields, like electric fields and magnetic fields, which makes them much more difficult to recycle. Therefore, novel and efficient processing technologies—collectively called post-shredder technologies (PSTs), such as the Volkswagen-SiCon process—are urgently required [7,12]. 

In the last decade, contactless sorting methods by means of sensor-aided sorting have been developed, collectively referred to as ‘Sensor-Aided Sorting’ or ‘Sensor-Based Sorting’. Liu [13] used thermogravimetric Fourier transform infrared spectrometer (TG-FTIR) to investigate the pyrolysis profiles of four plastics in ELVs and found that the Starink method was the most suitable for the separation of the blends. Kassouf [14] combined mid-infrared (MIR) spectroscopy with independent component analysis (ICA) to separate five commonly used plastics in vehicles and achieved 100% discrimination rates. Bezati [15] used X-ray fluorescence (XRF) spectrometry for the identification of polypropylene (PP) plastics in ELVs by using rare earth oxides as tracers; five of the seven tracers, tested with a 1 min exposure time at a concentration level of 1000 ppm, could be detected. Rozenstein [16] proposed a new method called Midwave Infrared (MWIR), which was useful for the sorting of colored and transparent plastics but it had the disadvantage of a long process time. Yan [17] investigated four handheld spectrometers based on different monochromator principles for the sorting of five of the most commonly used polymers, and a suitable analytical tool, SIMCA, was applied for the correct assignment polymer types. These technologies are able to separate most kinds of plastics efficiently but do not include the sorting of black or dark dyed plastics, which absorb most of the optical and NIR radiations and thus do not produce reflections for spectroscopic analysis [16]. XRT and MWIR methods can circumvent the effects of color, but they need too long an exposure time that is not feasible in industry. In addition to this, plastics that are used in vehicles are commonly modified by producers with their own preparations to optimize their mechanical properties or save the costs, and these additives are always kept secret. Thus, ELV recycling companies cannot obtain accurate data for calibrating their sensors and identification devices. 

In our previous studies, we proposed a novel method using laser-triangulation imaging together with frequency response recognition of impact acoustics for recycling ASR polymer scraps, and we proved its feasibility [18,19]. Impact acoustic technology has been widely used in the food industry over the last decade [20,21,22]. Objects with different particle sizes, shapes, or structures generate distinct AE signals. After automatic crushing, the majority of the plastic scraps in ASRs is flake-shaped and can be considered a two-dimensional (2D) structure with a thickness. In impact acoustic technology, the mechanical and shape parameters of scraps are the primary factors for determining the characteristics of specific kinds of materials, which means that the presence of unknown material blends and black and dark dyed materials are no longer obstacles. 

**Figure 1:**
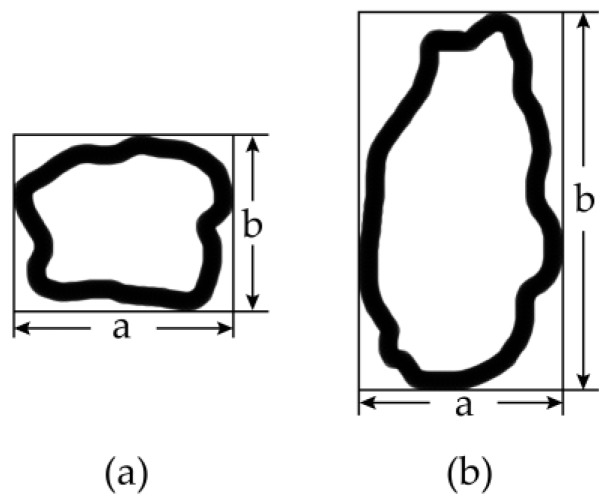
Scrap shapes: (**a**) regular shape; and, (**b**) irregular shape.

The method that was used for equivalent particle size determination in our previous research was mechanical fine sieving, which is the most commonly used method for raw material processing and sorting. The scraps to be tested with impact were sieved into several fractions by using screens with different sieve mesh sizes by employing manual or mechanical vibration devices. The process is characterized by simple screening equipment and easy operation, so it is a common application. However, plastic scraps with random shapes are generated through the shredding process and several irregular scraps are inevitably produced. A diagram of two representative scraps are illustrated in Figure 1. The width *a* and length *b* of scrap (a) are basically the same, while the dimensions of scrap (b) differ greatly, which means that the shape of scrap (a) is much more regular than (b). If the length-to-width ratio of a scrap, i.e., the value *b* divided by *a*, is larger than 1.68, the scrap is considered to possess an irregular shape [23]. The sieving method is effective for the measurement of scrap (a), with appropriate deviations, but for scraps with high irregularity, such as (b) illustrates, the deviations from using mechanical sieving could be very high. If irregular scraps like (b) are produced in high proportions, then this mechanical sieving method will become impractical, as the majority of the measurements are likely to be deviations. 

The screen mesh is also a part of the mechanical sieving process, and its sieving hole cannot be designed with high precision; typically, it can only be adjusted to within 1 mm. In our previous study, we proved that this accuracy is inadequate for distinguishing between pure and composite ASR plastics [18]. Moreover, mechanical sieving consumes too much time and energy [24], when considering that they are not being applied to separating scraps with high precision. Hence, the sorting efficiency of this method is strongly limited. 

Benefiting from the development of optic sensor technologies, such as digital image processing, computer vision, and digital photogrammetry, image measurement technology has been practically applied in many fields over the past decades, leading to great enhancement in efficiency and quality [25,26,27]. Image measurement refers to the method of measuring the characteristics and geometry of an actual object by using its image as a tool for morphological information. In this work, three-dimensional (3D) imaging measurements were employed instead of mechanical sieving for the computation of equivalent particle sizes of ASR scraps. The method performs with high accuracy and it has great flexibility. In particular, circularity analysis was found to significantly improve the sorting efficiency of pure and composite ASR plastics: acrylonitrile-butadiene-styrene (ABS) and its composite acrylonitrile-butadiene-styrene/polycarbonate (ABS/PC) and polypropylene (PP) and its composite polypropylene/ethylene-propylene-diene-monomer (PP/EPDM). 

## 2. Materials and Methods

### 2.1. Tested ASR Plastics

**Figure 2:**
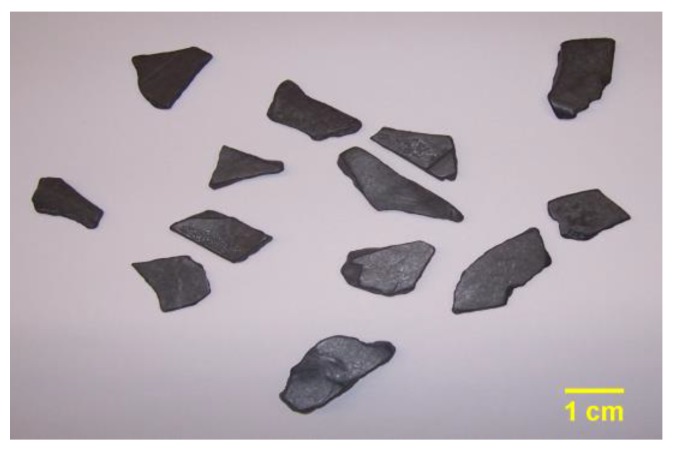
Samples of vehicle polymer materials.

ABS and PP as well as their composite materials ABS/PC and PP/EPDM are commonly used plastics in the vehicle industry. ABS and ABS/PC are often used in vehicle components, such as bumpers, dashboards, interior trim, and exterior trim; PP and PP/EPDM are usually used in fuel systems, body panel bumpers, under-bonnet, and electrical components [6]. The samples were collected from the products of an ASR plastic recycling company and had been crushed by a shredder into particle sizes of more than 200 mm. For this experiment, further crushing was needed, and all of the samples were crushed again in the laboratory through a shredder with a motor power of 37 kW running at 1475 r/min with a screen size of 23 mm. After crushing, the scraps were filtered by a sieve with a screen size of 13 mm. Some examples of the crushed scraps are shown in Figure 2.

### 2.2. Scraps’ Regularity Analysis by Using RRSB Distribution

As we mention in the introduction, during the crushing process, scraps with an irregular shape are generated randomly (Figure 1b). If there are too many scraps with irregular shapes, then the above-mentioned method is not appropriate for this research. Therefore, we need to determine the homogeneity of the scraps’ particle sizes. Normally, this determination is realized by studying the mass distribution according to the scrap sieving fractions with different particle sizes. 

In the sieving analysis, the masses of crushed scraps passing through each screen are expressed as the percentages of the total weight of sample. The interpretation of scraped samples of plastic materials generated from high speed impacts in shredder, spallation, and fragmentation process occur randomly. Many distribution models have been proposed to determine the homogeneity of the particle-size distribution (PSD) of crushed scraps [28,29]. One of the most important method is the Rosin–Rammler–Sperling–Bennet (RRSB) distribution, which is also called the Weibull distribution [30,31], which is usually used for describing the characteristics of the cumulative distribution of scrap sieving fractions. 

For crushed tested plastic materials. The cumulative masses of all sieving fractions with diameters smaller than *d* is defined as [24,30,31]:

(1) $$D=1-e^{-{\left(d/d\prime \right)}^{n}}$$

where *D* (wt %) is the passing rate of scraps corresponding to each particle size; *d* (mm) is the equivalent particle size of scraps; and, *d*’ (mm) is *d* when *D* = 63.2%. *d*’ is a statistical value that means that 63.2% of all scraps’ equivalent particle sizes are smaller than *d*’. The parameter *n* is used to describe the spread of the distribution. 

The RRSB function can be transformed, as follows:

(2) $$ln(1-D)=-{\left(d/d\prime \right)}^{n}$$

(3) ⇒          lg[−ln(1−D)]=nlg(d/d′)

(4) ⇒          lg[−ln(1−D)]=nlgd−nlgd′

Equation (4) can be further linearly defined as:

(5) $$y=nx+m\;,\;\;\;\;\;\;\;where\left\{ \begin{array}{l} y=lg\left[-ln\left(1-D\right)\right]\\ x=lgd\\ m=-nlgd\prime \end{array}\right.$$

Equation (5) is the statistical model of the RRSB function. According to the formulas of *x* and *y*, the specific values of the coordinate points [(*x*_1_, *y*_1_), (*x*_2_, *y*_2_), …, (*x_n_*, *y_n_*)] can be computed easily with experimental data. The values of *n* and *m* in equation (5) can be determined by putting the known coordinate points into a linear regression calculation. 

After the determination of Equation (5), the coefficient of regression is calculated, which is used to evaluate how well the data fit to each other. It is defined as:

(6) $$R^{2}\equiv 1-\frac{S_{er}}{S}\equiv 1-\frac{\sum {\left(y_{i}-{\hat{y}}_{i}\right)}^{2}}{\sum {\left(y_{i}-{\bar{y}}_{i}\right)}^{2}}$$

where *S_er_* is the residual sum of squares, *S* is the total sum of squares, *ȳ_i_* is the mean value of *y_n_*, and *ŷ_i_* is the associated modeled value of *y_n_*. *R*^2^ is a value that ranges from 0 to 1.0: *R*^2^ approaching 1.0 means that the data fit well to each other. According to the mechanism of RRSB distribution analysis, if there are certain amount of scraps with irregular shape, the mass variations between different sieving fraction would be relatively high, which leads to the passing rate distribution on *x* to be far from fitted line *y*, which were introduced in Equation (5), and this kind of deviation would result in the low value of *R*^2^ of passing rates. Therefore, if *R*^2^ is close to 1.0, it means that the particle sizes or masses are regularly distributed.

### 2.3. Impact Acoustic Sorting Theories

Scraps with different particle sizes or shapes generate different impact acoustics signals when an impact is applied. The key factors that decide the impact frequency response are the particle size and the thickness of the scraps, as verified in our previous work [18,19]. The mechanism of impacted acoustic frequency response was verified to be [18]:

(7) $$f=k_{C}\frac{h}{b^{2}},\;\;\left(k_{C}=\alpha_{m,n}\sqrt{\frac{E}{\rho \left(1-v^{2}\right)}}\right)$$

where *E* is Young’s modulus; *ρ* is the material density; *ν* is the Poisson’s ratio of the tested plastic material; *h* is the flake scrap thickness; *b* is the equivalent radius, i.e., the tested scraps’ particle size; *m*, *n* are integers (beginning with zero); and, *α_m,n_* is a dimensionless coefficient associated with the corresponding flexural vibration mode (*m*, *n*), which depends on different modes of impact, i.e., the impact position and shape of impact bodies. By impact tests and fitting analysis, the specific impact frequency coefficient *k_c_* of each material can be determined, which can then be used as a sorting criterion of different kinds of plastics. 

In our previous work, laser triangulation was used for the determination of the thickness of scraps, because it is a quick and accurate method. In this study, while we measured the thickness, the circularity was simultaneously determined using laser triangulation. 

**Figure 3:**
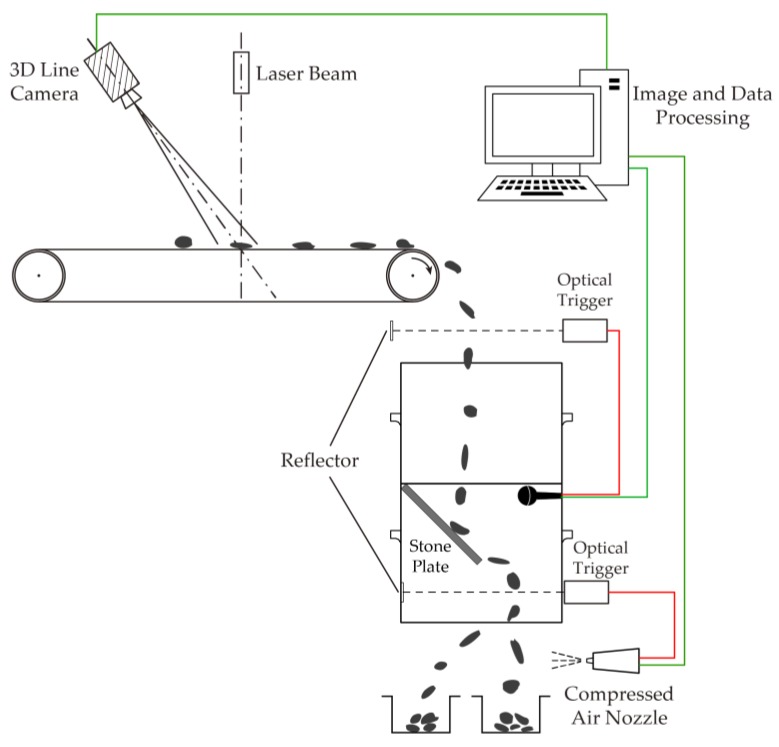
Experimental facility installation.

The installation of the whole experimental system, which is shown in Figure 3, includes the laser triangulation system and impact acoustics acquisition instrumentation. In this experiment, scraps were dropped from a conveyor to impact a stone plate, and an acoustic pickup was used to acquire the impact acoustics, which were then transferred to a computer. The laser triangulation system comprises a laser beam and a 3D line camera, which is used for the thickness measurement through image acquisition. Modern 3D line camera products have the ability to measure 3D visual parameters, positions, and morphological characteristics of objects synchronously. Its line rates are high, reaching 47,000 lines/s, and the resolution of the acquired image is 4096 × 8560 pixels. Finally, all the data were compiled in a computer for processing and analysis, and then a compressed air nozzle was controlled to sort the samples. This mechanism was introduced in detail in our previous studies [18,19]. 

### 2.4. Image Processing

**Figure 4:**
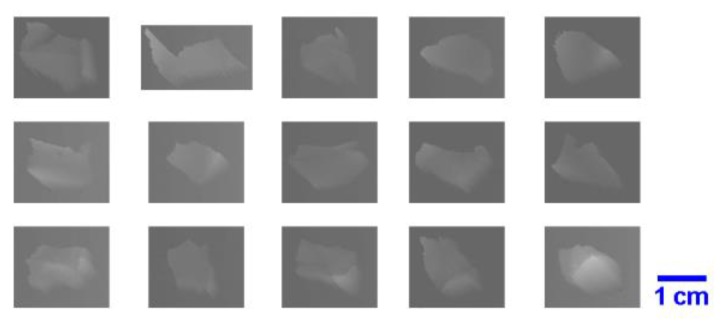
Three-dimensional (3D) grayscale images of tested objects.

The aim of image processing is to detect, locate, and segment the scrap images by border line recognition of each scrap image. By using a 3D line scan camera, scraps were imaged in grayscale; some examples are shown in Figure 4. In this research, all of the image processing and measurement works were performed automatically through programming while using MATLAB. 

**Figure 5:**
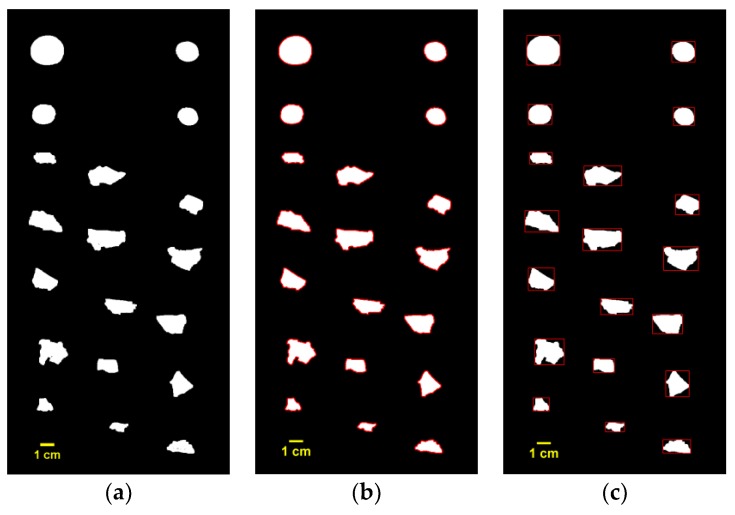
Image processing: (**a**) binary image; (**b**) image with border lines; and, (**c**) scraps with minimum rectangle bounding box.

The first step was to transform the grayscale images into binary images using grayscale threshold processing. In a binary image, all pixels contain only full black or full white values, i.e., 0 or 1. The transformation mechanism between the grayscale image and binary image is defined by:

(8) $$T_{ij}=\left\{ \begin{array}{l} 0\;\;\;\;\;\;\;\;\;\;(T_{ij}<T*)\\ 1\;\;\;\;\;\;\;\;\;\;(T_{ij}\geq T*) \end{array}\right.$$

where *T_ij_* is the grayscale value of pixels, a grayscale value of 0 means the pixel is totally black, and a grayscale value of 1 means that it is totally white; T* is the automatic threshold level calculated by using the filter function proposed by Otsu [32], which was used for its simple and convenient processing. The binary image is shown in Figure 5a. 

After binary transformation, small holes often appeared inside the scraps; therefore, a closed morphology operation was needed. A closed operation means expansion followed by corrosion, which has the effect of filling the small holes in the object, connecting adjacent objects, and smoothing the border. 

For the acquisition of scraps’ border lines, edge detection processing was used. Determination of border lines from a binary image is a standard problem in edge detection, and various methods have been proposed [33]. The Canny edge detector is an operator that uses a multistage algorithm to detect edges, and it is widely used due to its robustness to various conditions [34]. Subsequently, the extracted boundary lines were superimposed over the binary image, as shown in Figure 5b. 

According to scraps’ border lines, the smallest rectangle containing the scrap was determined for the segmentation of each scrap. Afterwards, we combined the rectangles with the original image, and the positional information of each scrap was determined, as shown in Figure 5c.

### 2.5. Circularity Determination

#### 2.5.1. Methods for Circularity Determination 

There are several normally used methods for circularity determination, such like Form factor method, Roundness factor methods, Radius ratio method, and Mean roundness method. 

**Figure 6:**
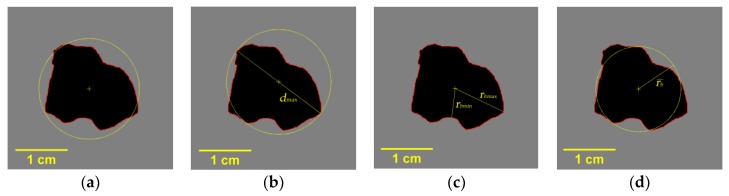
Diagrams of four circularity determination methods: (**a**) Form Factor; (**b**) Roundness Factor; (**c**) Radius Ratio; and, (**d**) Mean Roundness.

The Form Factor (FF) was first described as “percentage roundness” [35], which is defined by the American Society for Testing and Materials (ASTM). It is the most commonly used method and it can be described as the ratio of the object area to the area of a circle with the same perimeter, but it is usually calculated by using the perimeter itself, which is shown in Equation (9) [35]. The diagram in Figure 6a illustrates the basic principle of this method.

(9) $$FF=\frac{A}{A_{P}}=\frac{4\pi A}{P^{2}}$$

where *A* is the area of the target object, *A_P_* is the area of a circle with the same perimeter as the object, and *P* is the perimeter of the object. 

Another circularity determination method that is recommended by ASTM is called the Roundness Factor [36,37,38,39,40]. It was first presented by Campaña [39]; It is described as the ratio of the object area to a circle with the object’s maximum diameter, which is shown in Figure 6b.

(10) $$RF=\frac{A}{A_{dmax}}=\frac{4A}{\pi d_{max}^{2}}$$

where *A_dmax_* is the area of a circle with the maximum diameter of the object, and *d_max_* is the maximum distance between two random border points of the object. The greatest advantage of this method is that it avoids calculating the perimeter, thus eliminating a source of unnecessary errors.

The Radius Ratio (RR) was proposed by Ritter [40] and it is based on the definition of a circle. As the diagram in Figure 6c shows, it is defined as the ratio of the minimum radius to the maximum radius of the object.

(11) $$RR=\frac{r_{bmin}}{r_{bmax}}$$

where *r_bmin_* is the minimum radius from the border to the center point of the object and *r_bmax_* is the maximum radius. 

The Mean Roundness (MR) is based on the theory of the mean deviation. Its calculation accumulates the absolute differences between the radius of each border point and the average radius and then averages the accumulated value as the circularity [40]. Figure 6d is a sketch of this method.

(12) $$MR=\frac{1}{n}\sum_{j=1}^{n}\frac{\bar{r_{b}}}{\left|r_{j}-\bar{r_{b}}\right|+\bar{r_{b}}}$$

where *j* is the border point of the object, *r_j_* is the corresponding radius from the border point *j* to the center point, and *r_b_* is the mean radius from the border points to the center point of the object. 

#### 2.5.2. Calculation of Scrap Shape Parameters

During the circularity calculation, scrap shape parameters, such as perimeter, area, and centroid are used, which are described in the following paragraphs.

**Figure 7:**
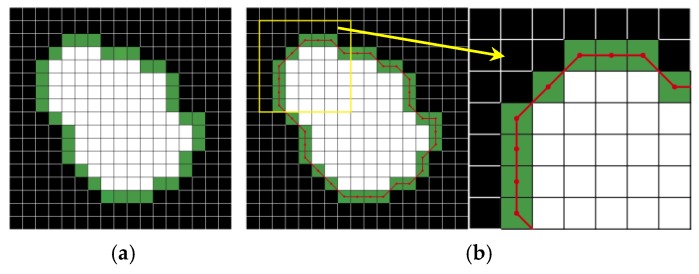
Border of an object: (**a**) border pixels of the object; and, (**b**) border line of the object.

The perimeters are computed by calculating the distance between each adjoining pair of pixels around the border of the object. The green pixels in Figure 7a illustrate the border pixels included in the perimeter calculation for this object. The border line thus obtained is a combination of lines between the centers of two adjacent border pixels, as the red line shows in Figure 7b. The perimeter is calculated according to the following equation [38]:

(13) $$P=\sum_{j=1}^{n-1}D\left(b_{j},b_{j+1}\right)$$

where *b_j_* and *b_j_*_+1_ are the adjacent border pixels and *D* equals 0.948 when *b_j_* and *b_j_*_+1_ are on a straight line; otherwise, *D* equals 1.343, which has been confirmed by Niehaus [41].

Since the border line cuts each border pixel into two parts, the area of the object changes correspondingly and it can be computed by using:

(14) A=a+0.5b

where *a* is the number of pixels inside the border and *b* is the number of border pixels.

The other parameter needed in the measurement is the centroid. The quality center is referred to as the centroid, which specifies an imaginary point on the material system where the quality is considered to be concentrated. In this research, all ASR scraps are considered to have a uniform density. Therefore, the centroid is computed by [42]:

(15) $$\left\{ \begin{array}{l} c_{x}=\frac{1}{6A_{s}}\sum_{i=0}^{n-1}\left(x_{i}+x_{i+1}\right)(x_{i}y_{i+1}-x_{i+1}y_{i})\\ c_{y}=\frac{1}{6A_{s}}\sum_{i=0}^{n-1}\left(y_{i}+y_{i+1}\right)(x_{i}y_{i+1}-x_{i+1}y_{i}) \end{array}\right.$$

where (*c_x_*, *c_y_*) define the centroid of the object, (*x_i_*, *y_i_*) are the pixels of the object, and *A_s_* is the signed area of the object, which can be obtained by [42]:

(16) $$A_{s}=\frac{1}{2}\sum_{i=0}^{n-1}\left(x_{i}y_{i+1}-x_{i+1}y_{i}\right)$$

As the unit of the parameters acquired from the image is a pixel, the conversion factor k was used, which is defined as the number of pixels of the image width and the actual width of the scanning area, which can be acquired by:

(17) $$k=\frac{d_{actual}}{d_{pixel}}$$

where *d_actual_* is the actual width of the scanning area with a unit of mm, *d_pixel_* is the number of pixels occupied by the horizontal direction of the image with a unit of pixel, and the unit of *k* is mm/pixel. 

## 3. Results and Discussion

### 3.1. Results of Regularity Analysis

**Figure 8:**
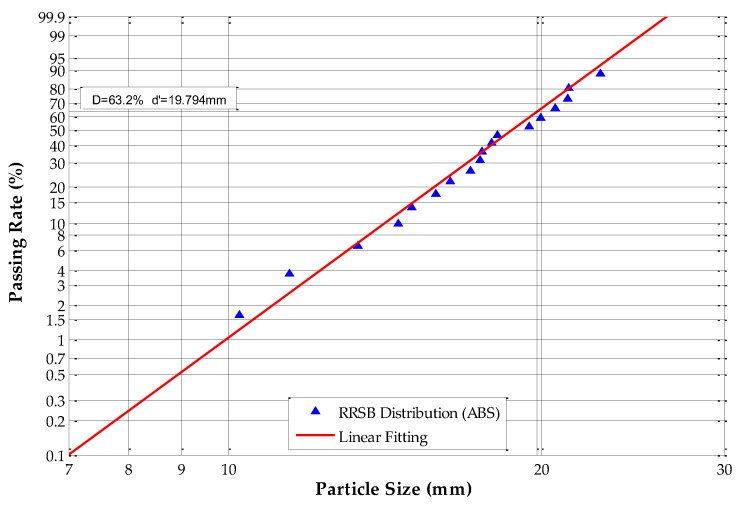
Rosin–Rammler–Sperling–Bennet (RRSB) distribution of acrylonitrile-butadiene-styrene (ABS) scraps.

**Figure 9:**
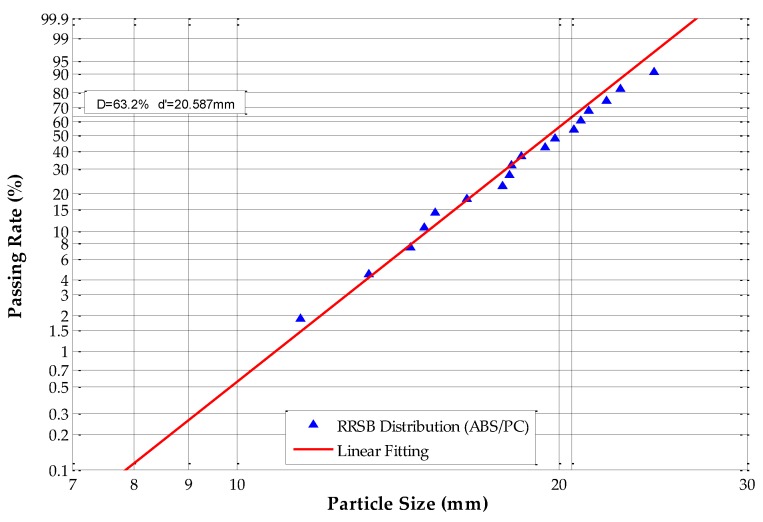
RRSB distribution of acrylonitrile-butadiene-styrene/polycarbonate (ABS/PC) samples.

**Figure 10:**
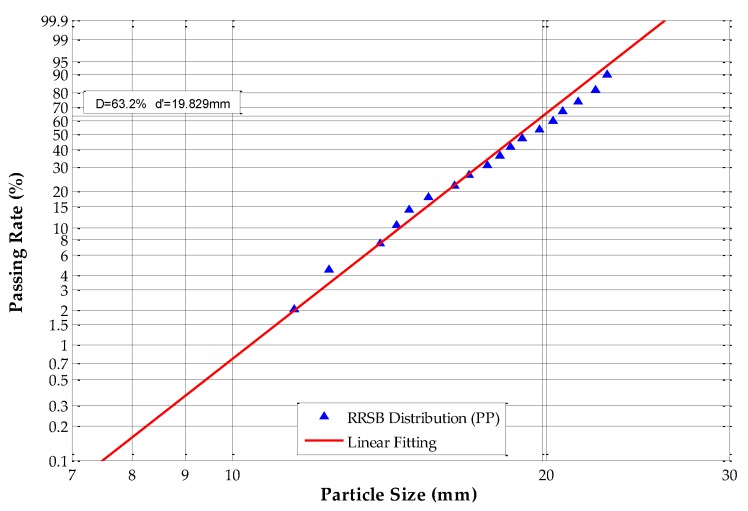
RRSB distribution of polypropylene (PP) scraps.

**Figure 11:**
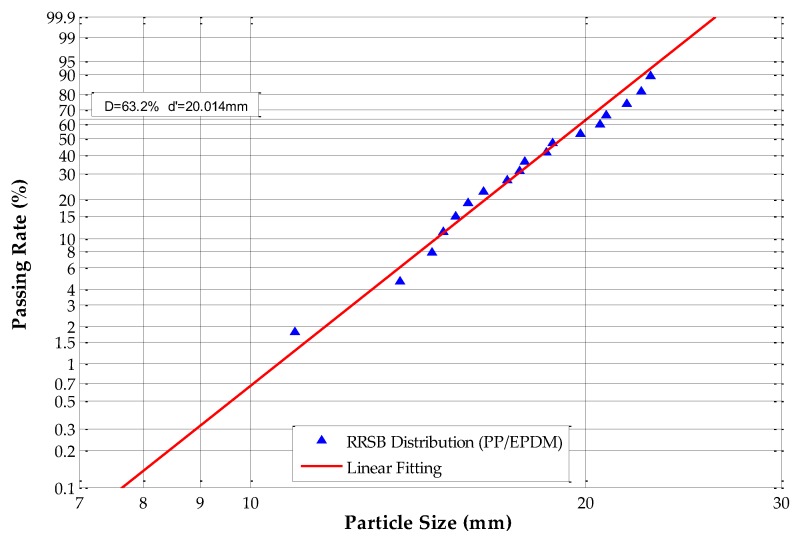
RRSB distribution of polypropylene/ethylene-propylene-diene-monomer (PP/EPDM) scraps.

The equivalent particle sizes were analyzed and calculated according to Equations (10) and (17); in this paper, *k* is equal to 0.305. The Rosin–Rammler–Sperling–Bennet (RRSB) size distribution and fitting analysis results are shown in Figure 8, Figure 9, Figure 10 and Figure 11. 

Figure 8 shows that, for ABS scraps, *d’* = 19.794 mm and the passing rate is 63.2%, which means that 63.2% of the tested ABS scraps have an equivalent particle size of under 19.794 mm. The slope *n* of the fitting line equals 7.2776, and *R*^2^ equals 0.9924. The RRSB distribution analysis of ABS/PC scraps shows that *d’* = 20.587 mm and the passing rate is 63.2%, which means that 63.2% of the tested ABS/PC scraps have an equivalent particle size under 20.587 mm. The slope *n* of the fitting line equals 8.8396, and *R*^2^ equals 0.9937. The RRSB distribution analysis of the PP samples shows that *d’* = 19.829 mm and the passing rate is 63.2%, which means that 63.2% of the tested PP scraps have an equivalent particle size under 19.829 mm. The slope *n* of the fitting line equals 8.7075 and *R*^2^ equals 0.9886. The RRSB distribution analysis of the tested PP/EPDM scraps shows that *d’* = 20.014 mm and the passing rate is 63.2%, which means that 63.2% of PP/EPDM samples have an equivalent particle size under 20.014 mm. The slope *n* of the fitting line equals 7.9348, and *R*^2^ equals 0.9830. 

The results demonstrate that all four types of samples have fitting coefficients (*R*^2^) that are very close to 1.0, which means that the PSD of these four scrap materials had adequate homogeneity and regularity. The occurrence of irregular scraps was very low, which fulfills the requirements for circularity determination.

### 3.2. Determination of Scrap Circularity

**Figure 12:**
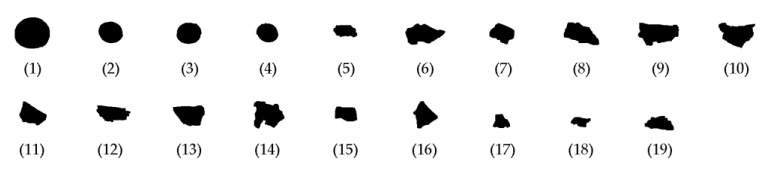
Scrap images acquired during testing.

**Figure 13:**
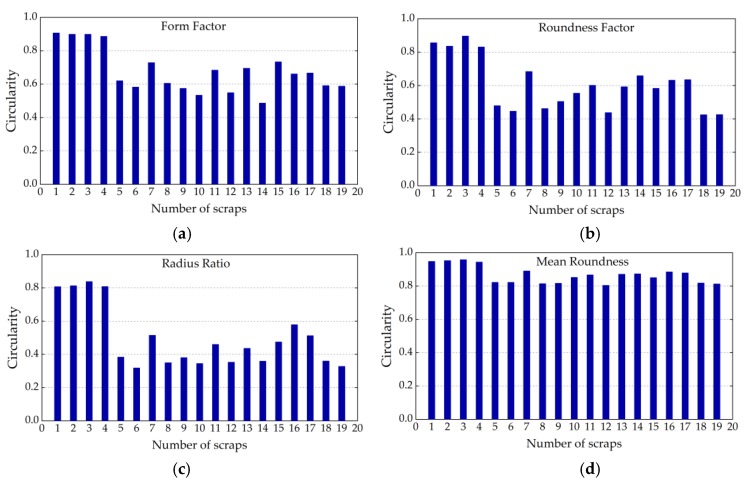
Bar plots of measurement results: (**a**) Form Factor; (**b**) Roundness Factor; (**c**) Radius Ratio; and, (**d**) Mean Roundness.

According to the verified regularity of tested polymers scraps, the above-mentioned circularity measurement algorithms were implemented using MATLAB. The objects in the processed images were identified and their circularities were measured. The results of all the circularity determination methods are within the range (0, 1], and a value is equal to 1 if, and only if, the measured object is a perfect circle. Some tested plastic scraps are shown in Figure 12 as examples, and the circularity calculation results of the four above-mentioned methods are shown in Figure 13. In addition to the tested samples, we added four nearly circular scraps as references, which are the scraps numbered 1–4 in Figure 12.

**Figure 14:**
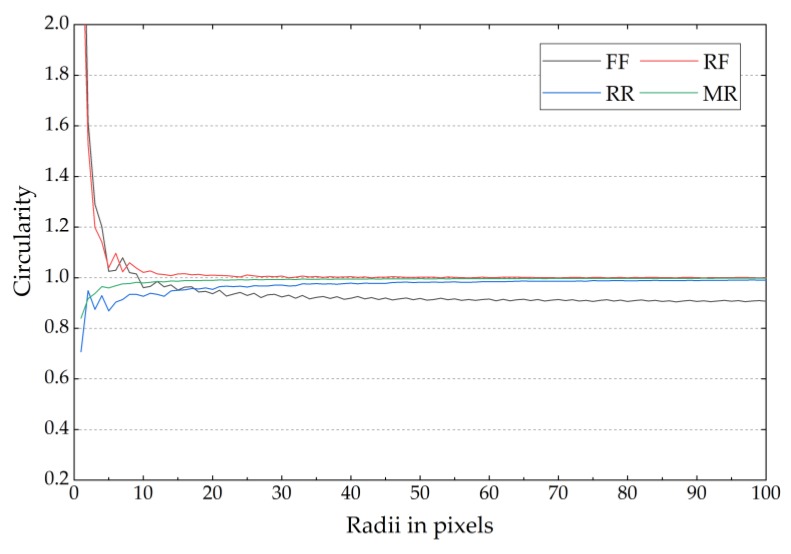
Circularities of perfect circles with varying radii.

In order to analyze the precision of the four methods in this research, perfect circles were artificially programmed with varying radii, ranging from 1 to 100 (in pixels), and tested. The results are shown in Figure 14. As can be seen from the figure, the Form Factor and Roundness Factor methods do not satisfy the basic requirement that the calculated circularity must have a range of (0,1]. In other words, the Form Factor and Roundness Factor are more sensitive to resolution than the other two methods. 

**Figure 15:**
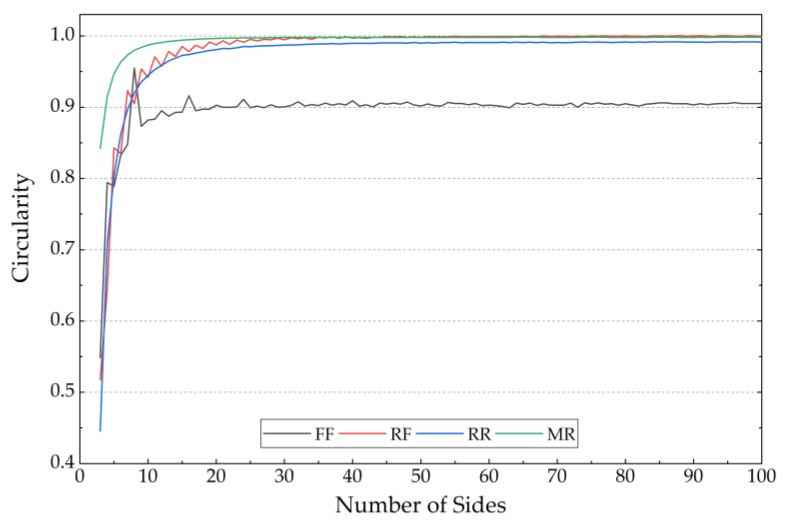
Circularities of regular polygons with varying sides.

For a regularity analysis of the tested scraps, a simulated polygon method was programmed and tested on polygons with sides ranging from 3 to 100. The results are shown in Figure 15, where it can be seen that as the number of sides’ increases, the circularity approaches 1.0 correspondingly. However, as opposed to the other three methods, the Form Factor method (FF) only reaches 0.9, which has been verified as unreliable for quasi-circular objects. 

From Figure 13, Figure 14 and Figure 15, it can be concluded that the Form Factor method is easily affected by image resolution and it performs very poorly for a circle; also, the inaccuracy of the object perimeter calculation is likely to produce the most deviations in the results. The Roundness Factor method is also affected by low resolution, but it bypasses the perimeter calculation, which can reduce measurement errors, and it performs well when measuring irregular scraps. Another factor that has an impact on the Roundness Factor is the occurrence of burrs, which can cause errors in the determination of the maximum diameter. However, in crushed plastics, it is a seldom occurrence and it can be neglected. For the Radius Ratio method, generally, the resulting circularities are much lower than those from the other three methods, as its maximum value is only 0.8381. For scraps with both depressions and protrusions, the result will be affected greatly, and these aspects commonly appear in crushed plastics. For the Mean Roundness, although it is very applicable to the circularity determination for scraps that approximate a perfect circle, this metric does not take into account the effect of the maximum diameter of the scraps, which normally does not cover the entire area of the tested scraps. Thus, this can lead to a leakage effect of the impact frequency response since, with the mean roundness method, not all vibrating parts on the tested scraps are included in their equivalent circularities. This deviation could introduce large errors to the recognition criterion described in Equation (7). The Roundness Factor does not have this problem because, according to Equation (10), the entire area of all the tested scraps are included in the equivalent circularity. Hence, in this research, we chose the Roundness Factor method to determine the circularities of the tested scraps. 

When considering that the resolution of the acquired images is high (4096 × 8560 pixels), regular scraps occupied the highest proportion of the ASR scraps. Therefore, in this research, the Roundness Factor method was used for the scraps’ circularity calculation, and the equivalent radius was determined as half of *d_max_* in Equation (10). The final results are shown in Table 1.

### 3.3. Results of Sorting Efficiency Optimization

**Figure 16:**
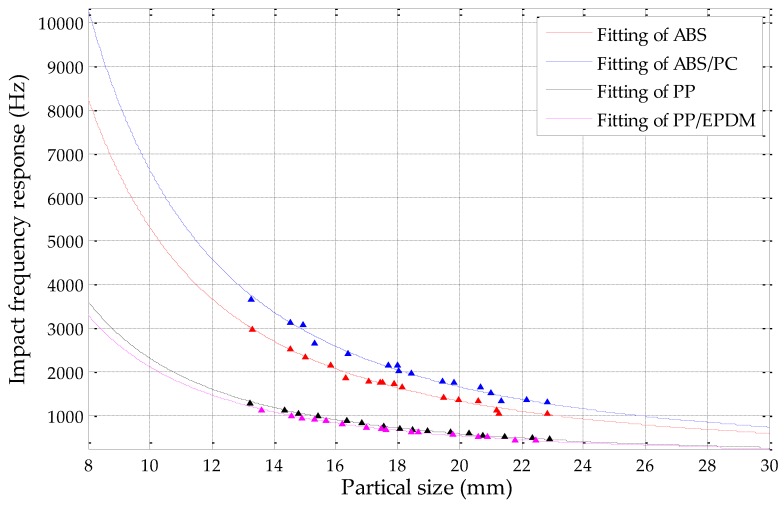
Fitting analysis of four samples’ impact frequency response.

After the determination of equivalent particle size, a fitting analysis of the impact frequency response for the four kinds of scraps was implemented by using the mechanism demonstrated in Equation (7), as well as our previous studies. The analysis used scraps with a particle size greater than 13 mm (not inclusive) and smaller than 23 mm, as particles with sizes that are in this range can generate acoustic signals with adequate intensity. The fitting results of ABS, ABS/PC, PP, and PP/EPDM are shown in Figure 16. 

Figure 16 shows that the impact acoustic frequency response distributions of polypropylene (PP), polypropylene/ethylene-propylene-diene-monomer (PP/EPDM), as well as acrylonitrile-butadiene-styrene (ABS) and acrylonitrile-butadiene-styrene/polycarbonate (ABS/PC), according to the circularity determined equivalent diameter. The impact frequency coefficient *k_C_* was fitted with high accuracy. 

The *k_C_* of ABS is 530.25 m/s, and the fitting coefficient is 0.9874; the *k_C_* of ABS/PC is 661.74 m/s, and the fitting coefficient is 0.9874; the *k_C_* of PP is 231.47 m/s, and the fitting coefficient is 0.9952; the *k_C_* of PP/EPDM is 211.77 m/s, and the fitting coefficient is 0.9830. From Figure 16, it can be observed that the fitting curves of ABS-based materials are far from those of PP-based materials, meaning that, theoretically, they could be completely sorted. The *k_C_* values of ABS and ABS/PC are very close, which means that they have similar impact frequency response properties; PP and PP/EPDM are in a similar situation. Therefore, the coefficient *kc* of ABS-based and PP-based materials are similar, which limits their recognition accuracy and sorting efficiency. In our previous study, the actual sorting efficiency for the scrap materials with diameters ranging from 14 to 23 mm was 39.2% for PP and 41.4% for PP/EPDM scraps; similarly, it was 62.4% for ABS and 70.8% for ABS/PC scraps [18]. 

In this research, confidence interval analysis was applied to determine the sorting efficiency. Confidence level describes the frequency (i.e., the proportion) of confidence intervals that contain the true value of the studied parameter. In other words, if confidence intervals were built under a certain confidence level with adequate experimental data, then we could define the proportion of the true value of studied parameter that is contained in these intervals to match this confidence level. If we set the original hypothesis as the impact acoustic frequency response located in the confidence interval was true, and we might use *p* as the probability (significance level) of original hypothesis rejecting, then the confidence level could be defined as 1 − *p*.

**Figure 17:**
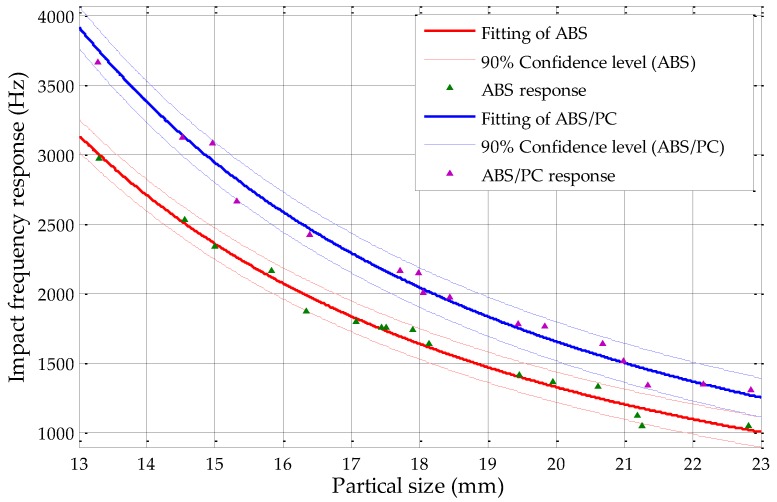
The 90% confidence level of the ABS and ABS/PC mixture by using circularity measurements.

**Figure 18:**
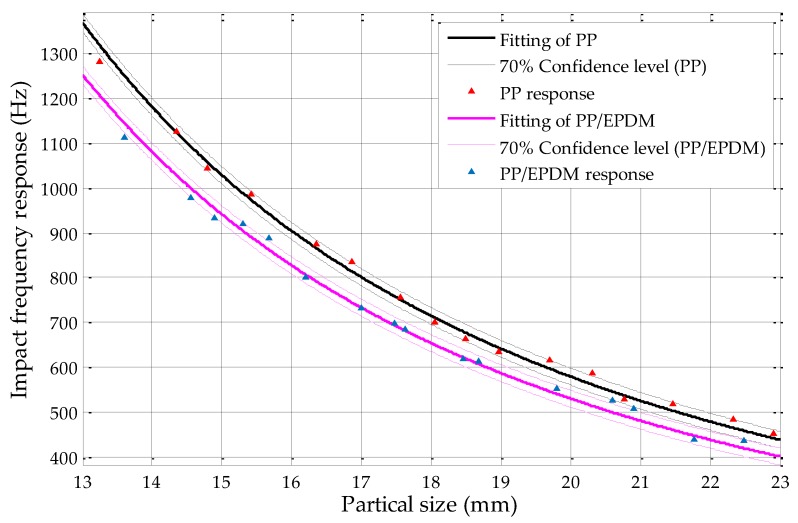
The 70% confidence level of the PP and PP/EPDM mixture by using circularity measurements.

Based on our previous work using fine sieving, the original confidence interval analysis results of the ABS and ABS/PC mixtures and the PP and PP/EPDM mixtures were 75% and 50% sorting confidence levels, respectively. Based on the results of this study using circularity measurements, the 90% sorting confidence level of the ABS and ABS/PC mixture and the 70% sorting confidence level of the PP and PP/EPDM mixture are shown in Figure 17 and Figure 18, respectively. In order to make the figure clear, not all of the responses are illustrated and the responses out of the confidence intervals are not demonstrated.

**Figure 19:**
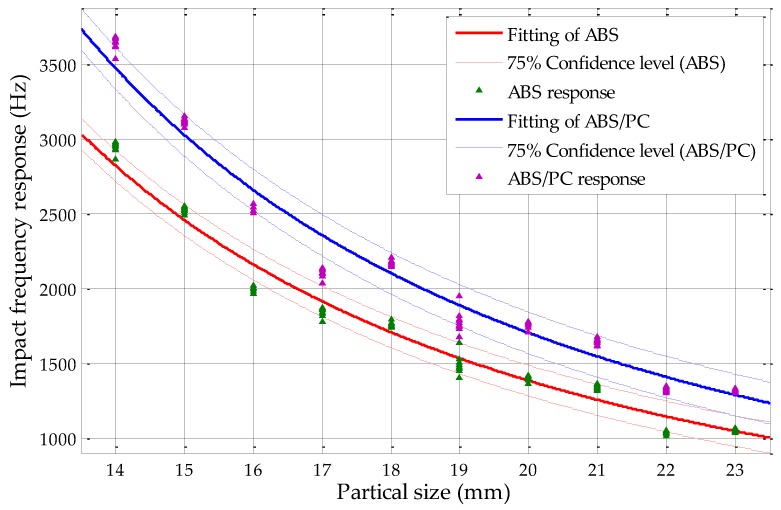
Confidence intervals of ABS and ABS/PC at the 75% confidence level by using fine sieving diameter determination.

From Figure 17, it could be found that without overlapping of confidence intervals, the maximum confidence level of ABS and ABS/PC mixtures by using circularity measurement could achieve 90%, with a particle size range from 13 to 23 mm. When compared with the results by using fine sieving determination in former studies which is shown in Figure 19, the fine sieving diameter determination method could only achieve confidence level of 75% without any overlapping of confidence intervals [18], which means that its maximum sorting efficiency can reach 75%; by using the circularity measurement which is introduced in this study, the sorting efficiency of the ABS and ABS/PC scraps could be optimized up to 90%. Relative to the sieving method, the sorting efficiency is increased by 15%, approximately.

**Figure 20:**
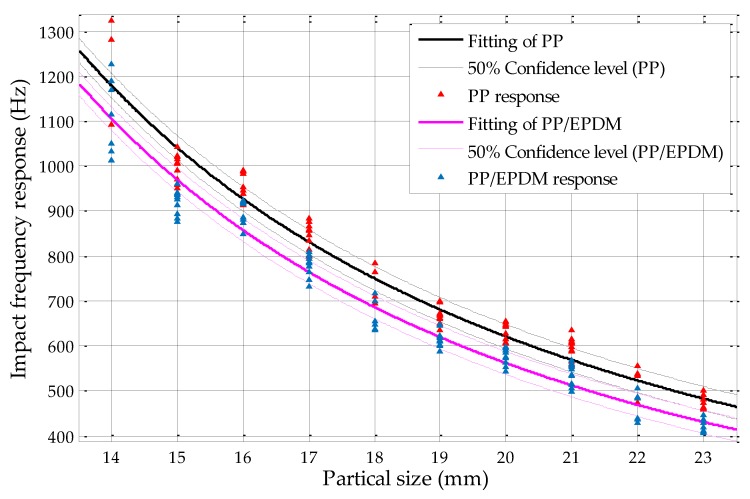
Confidence intervals of PP and PP/EPDM at the 50% confidence level by using fine sieving diameter determination.

From Figure 18, it can be found that, without overlapping of confidence intervals, the maximum confidence level of PP and PP/EPDM mixtures by using circularity measurements could achieve 70%, with particle sizes ranging from 13 to 23 mm. When compared with the results by using fine sieving diameter determination in former studies, which is shown in Figure 20, the fine sieving diameter determination method can only achieve confidence level of 50% without overlapping of confidence intervals [18]; by using the circularity measurement method, the maximum sorting efficiency of the PP and PP/EPDM scraps can be optimized up to 70%. Relative to the sieving method, the sorting efficiency is increased by 20%, approximately.

From the analysis of confidence level, it can be found that the distribution of impact acoustic frequency responses of tested vehicle plastic materials by employing the image measurement method is much more reasonable than the results from fine sieving method. Contrary to the fine sieving method, the equivalent particle sizes determination by photogrammetry using image circularity measurement can avoid the problem of discrete particle sizes’ determination with a step-length of min. 1 mm; the regularities of tested crushed plastic scraps were also confirmed by using the RRSB distribution analysis, which ensured the accuracy of the final fitting analysis and the confidence intervals. According to the results of this study, we can find that the acquired impact acoustic frequency responses had gotten closer to the fitting curves than before. The number of frequency responses which located in the non-overlapped confidence intervals had increased, hence the confidence level increased correspondingly. The new optimization procedures improved the sorting efficiency by 15% to 20%. 

## 4. Conclusions

In this study, individual scrap-oriented circularity measurement using a 3D imaging method was employed for the calculation of their circularities, i.e., the equivalent particle size of tested plastic scraps. Also, an RRSB distribution analysis was used to analyze the overall regularity of the tested scrap mixtures. According to accuracy analysis in this study, the Roundness Factor (RF) method was verified to be the most suitable for the determination of the scraps’ circularity. All of these experiments were conducted to optimize the sorting efficiency of automotive shredder polymer residues using the impact frequency response recognition method, which was developed and reported in our previous research in reference [18]. 

This follow-up study shows that by further processing and data mining of acquired 3D grayscale images with circularity measurements instead of mechanical fine sieving, the sorting efficiency for ABS and ABS/PC scrap mixtures approaches 90%, which is an increase of about 15% relative to the results of our previous study; for PP and PP/EPDM scrap mixtures, the sorting efficiency approaches 70%, which is an increase of about 20% when compared with the results of our previous research. The results illustrate that this new scrap size determination method using 3D imaging and circularity analysis can significantly optimize the sorting efficiency of ASR plastic materials, and it performs better than the original fine sieving method. Although complete separation is still not possible, the novel method notably improves the recognition efficiency and can thus produce secondary recycled materials with a much higher quality than before. With an emphasis on distinguishing between pure and composite plastic materials with very similar properties, this method has a significant higher efficiency relative to others. Meanwhile, this method of circularity determination could replace the traditional method of mechanical sieving and results of system simplification and energy conservation. In further research, more scrap shapes and other polymer materials need to be studied. 

## Figures and Tables

**Table 1:** Results of circularity determination.

No.	Circularity	No.	Circularity	No.	Circularity
1	0.8561	8	0.4621	15	0.5837
2	0.8361	9	0.5048	16	0.6321
3	0.8964	10	0.5540	17	0.6349
4	0.8311	11	0.6018	18	0.4252
5	0.4796	12	0.4376	19	0.4263
6	0.4464	13	0.5925		
7	0.6840	14	0.6592		
